# Demixing of four simultaneously co-expressed phase-separating proteins in the endoplasmic reticulum lumen

**DOI:** 10.1042/BSR20253165

**Published:** 2025-09-02

**Authors:** Haruki Hasegawa

**Affiliations:** Discovery Protein Science, Department of Large Molecule Discovery and Research Data Science, Amgen Inc., South San Francisco, CA, 94080, U.S.A.

**Keywords:** endoplasmic reticulum, protein phase separation, intracellular protein crystallization, protein droplet, inclusion body

## Abstract

Intracellular protein crystallization represents an intriguing form of biomolecular assembly. While the list of intracellularly crystallizing proteins is growing and their physiological roles are being elucidated, the underlying requirements and processes for intracellular crystallogenesis remain largely unknown. To reveal cellular capacity and morphological plasticity to accommodate protein crystals and crystal-like inclusion bodies, this study examines how simultaneously co-expressed phase-separating proteins influence each other’s behavior in the endoplasmic reticulum (ER) lumen. To this end, four cargoes were selected based on their ability to produce distinctive inclusion body types and morphologies irrespective of originating species, function, or sequence homology. The co-expressed model proteins independently phase-separated into distinctive inclusions and coexisted in the ER without losing their signature morphologic characteristics. The continued growth of intra-ER protein crystals and droplets suggested that co-expressed cargo proteins were continuously synthesized and folded in the ER, thereby fueling the growth of the corresponding inclusion bodies. Thus, even in the crowded ER environment, each of the four overexpressed cargo proteins can find their mates through self-association and assemble into four unique structures in the ER. This study demonstrates that cells can accommodate up to four distinct types of mesoscale inclusion bodies in the ER lumen simultaneously, and the respective phase-separation events proceed without interfering with each other and without morphological mixing.

## Introduction

Intracellular protein crystallization is an intriguing biomolecular assembly process. The earliest report on such an event was published in 1853 by Charcot and Robin [1] and then in 1872 by Leyden [2] for bipyramidal protein crystals detected in basophils and eosinophils (now called Charcot–Leyden crystals, or CLCs). Their finding was followed by Reinke’s 1896 report on the hexagonal prism-shaped crystals in testicular Leydig cell cancers [3] and by Glaus in 1917 on the immunoglobulin crystals found in multiple myeloma cells [4]. Since then, intracellular protein crystals have been described in many branches of life [5–7] and occur in humans under both physiological and pathological settings [5,8].

Compared with the process of membrane-less organelle formation by liquid–liquid phase separation (LLPS) [9,10], the underlying requirements for intracellular protein crystallization are still poorly understood [11–17]. However, as illustrated below, functional and physiological importance is beginning to be elucidated for various intracellular protein crystals across different cell types and organisms. First, because of dense packing and resistance to proteolysis, the crystalline state is employed as a space-efficient way to sequester functional proteins at high concentrations. This scheme is employed by insulins in pancreatic β-cells [18], eosinophil’s major basic protein-1 [19], lipoprotein crystals of yolk platelets in amphibian eggs [20], the endoplasmic reticulum (ER)-derived nutritional protein bodies of plants [21], and the insecticidal toxin crystals of *Bacillus thuringiensis* [22]. Secondly, intracellular protein crystals can function as solid-state catalysts. Three examples have been reported, and all three are peroxisome-localizing enzymes. The first is alcohol oxidase from methylotrophic yeast [23]. The second is urate oxidase from rat liver cells [24], and the third is catalase from sunflower cotyledon and potato tubers [25]. Thirdly, intracellular and *in vivo* protein crystals play roles in pathogenesis and disease diagnosis in [1] a group of proliferative diseases, such as multiple myeloma, plasmacytoma, myeloma, and lymphocytic leukemia [2,8]; crystal-storing histiocytosis [3,26]; cataracts [4,27]; hemoglobin C disease [5,28]; CLCs that trigger type 2 immunity pathogenesis, including asthma and chronic rhinosinusitis with nasal polyps [6,29,30]; neuromuscular diseases such as mitochondrial myopathies [7,31]; and Reinke crystals found in Leydig cell tumors [32]. Fourthly, intracellular protein crystals are adapted to fulfill the functional needs of diverse organisms in their unique living environments. For example, several terrestrial and marine bioluminescent organisms use protein crystallization as a shared approach to store enzymes in their light-emitting organs (see [17] and references therein). The fruit fly Drosophila uses specialized ‘crystal cells’ that house crystals of prophenoloxidase-2 for oxygen sensing, storage, and transport [33]. In filamentous fungi, *Neurospora crassa*, cells house unique organelles called Woronin bodies composed of HEX-1 protein [34] that seals the plasma membrane when cells are physically damaged [34,35]. The ciliated protozoan *Paramecium* stores crystalline secretory products called trichocysts that are discharged as a defense mechanism [36]. These examples clearly illustrate that natural selection exploits various strategies to enable protein crystallization inside the cells when advantageous for the cells or the organismal survival. Likewise, in certain disease settings, intracellular protein crystallization is induced during pathogenesis and causes deleterious symptoms. However, the degree of selection pressure favoring or disfavoring such protein crystallization in wildtype sequences and disease alleles varies considerably [37]*.*

Although intracellular crystallization of one specific protein seems challenging enough, cells may possess unexploited capacity to support the simultaneous crystallization of two or more different cargoes inside the cells, as previously shown both in recombinant [15,16] and natural [38] settings. While the cell’s surprising plasticity to accommodate rod-shaped protein crystals longer than 200 μm was revealed using a few different cargo proteins [16,17,39], the cell’s biosynthetic capacity and physical limits have not been evaluated regarding how many different types of inclusion bodies can form and coexist simultaneously in a given organelle. To examine how cells sort out the mixture of spontaneously phase-separating cargoes in a given subcellular compartment, four biologically unrelated model proteins were selected and co-expressed in different combinations using HEK293 cells. A key experimental requirement for this study is to find and select a right set of model proteins that produce completely different inclusion body morphology in the ER lumen. Thanks to the stark difference in inclusion body characteristics, it became clear that co-expressed four proteins simultaneously yet independently phase-separated into respective inclusion body types in the ER and stably coexisted without being merged, mashed up, or interfering with one another. The result asserts that proteins can phase-separate into crystals and droplets despite abundant bystander proteins and other overexpressed cargoes by selectively finding their mates.

## Hypothesis

Selecting evolutionarily and functionally unrelated, spontaneously phase-separating model cargoes that form distinct inclusion bodies enables the examination of how many different types of phase-separation events can occur simultaneously in a given organelle. This experimental approach demonstrates the biosynthetic capacity and morphological flexibility of cells, as well as provides insights into the regulation of protein mixing and demixing within an organelle.

## Results

### Selection of four model cargoes that spontaneously phase-separate into readily distinguishable inclusion bodies in the ER lumen

Transiently transfected HEK293 cells were previously shown to possess enough biosynthetic capacity to support at least four independent intracellular protein crystallization events taking place simultaneously in different subcellular compartments of a single cell [15]. To extend the previous findings, this study assessed whether four different proteins can phase-separate simultaneously in a single subcellular compartment of the cell. To this end, four model cargo proteins were selected based on the following three criteria. (i) Each model protein is already known to phase-separate into a distinguishable inclusion body in the ER lumen, and no new extensive validation is required. (ii) Each cargo protein generates inclusion bodies of consistent characteristics unique to the cargo protein, so the given protein phase-separation event is readily tractable and identifiable based on the inclusion body properties. (iii) The inclusion body characteristics of the four model proteins are different enough from each other, so the inclusion body shape or type can be used as a reliable identifier of the corresponding phase-separation events without any labeling.

**Figure 1:**
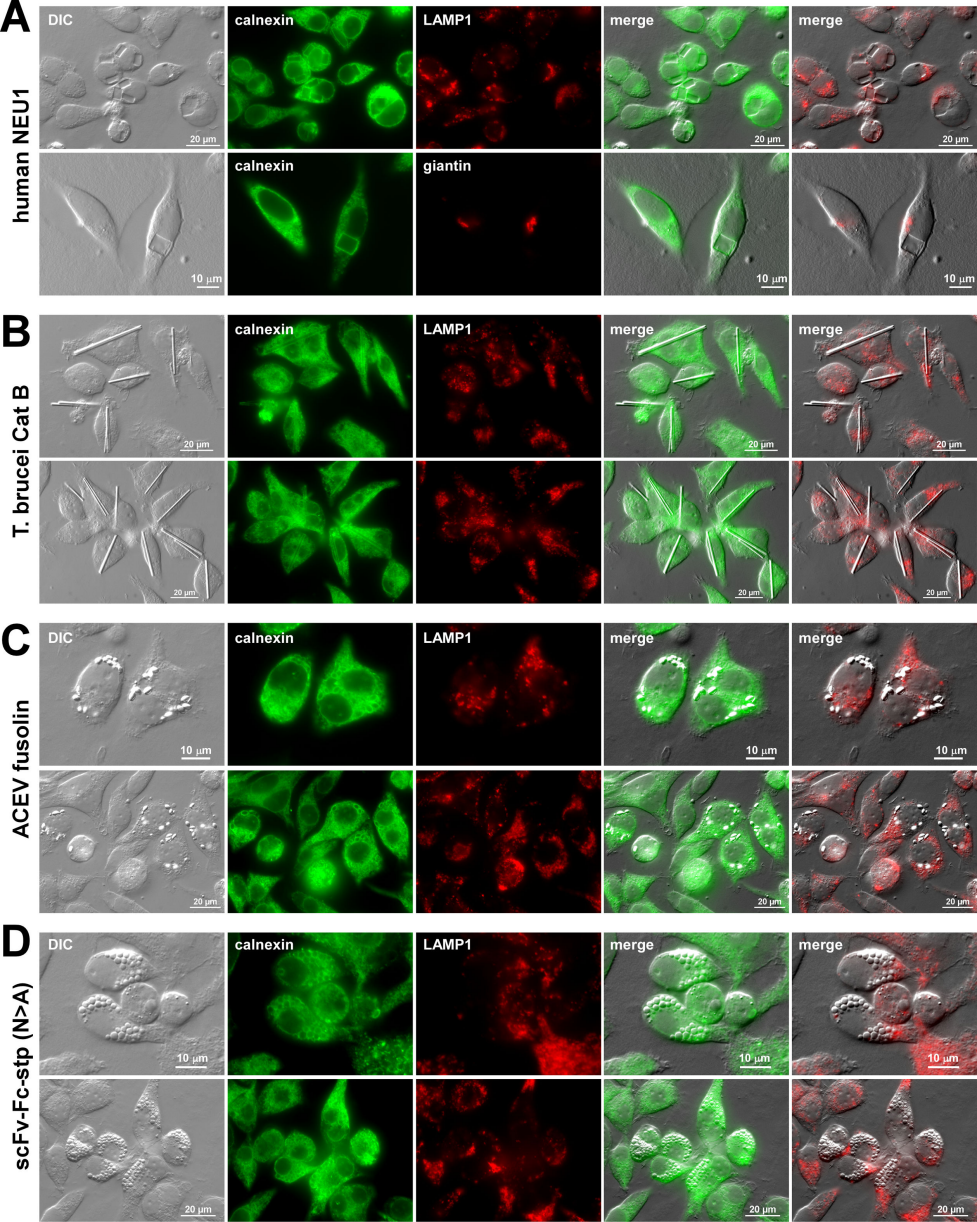
The selected four model cargo proteins produce characteristic and distinguishable inclusion body types in the ER lumen. Differential interference contrast (DIC) and fluorescent micrographs of HEK293 cells transfected to overexpress (**A**) human NEU1, (**B**) *T. brucei* Cathepsin B (Cat B), (**C**) ACEV fusolin, and (**D**) IgM-like oligomeric secretory protein, scFv-Fc-stp (*N*>A). On day-3 post-transfection, cells were fixed, permeabilized, and immunostained with rabbit anti-calnexin (shown in green). Co-staining was performed with mouse anti-giantin (shown in red, A, second row) or mouse anti-LAMP1 (shown in red, all the rest). Both green and red images were digitally overlayed with DIC to create ‘merge’ views.

The following four proteins satisfied the above-mentioned criteria. (i) Human NEU1 was chosen because it generates cubic and square plate-like protein crystals in the ER lumen in various mammalian cells, including HEK293 cells [15,40] (see Figure 1A). (ii) *Trypanosoma brucei* cathepsin B (Cat B) was selected because it crystallizes into rod-shaped inclusion bodies in the ER when overexpressed in Sf9 insect cells [41,42] and HEK293 cells (see Figure 1B). (iii) An insecticidal protein, fusolin, from *Anomala cuprea* entomopoxvirus (ACEV) was picked because it produces spindle-shaped crystals in the ER lumen of mammalian cells when recombinantly expressed (Figure 1C and [16]). Now, aside from cube-, rod-, and spindle-shaped crystals mentioned above, there was a dire need for the fourth cargo protein that produces yet another inclusion body morphology. Unfortunately, other recombinantly validated proteins known to crystallize in the ER tend to generate crystals with rod/needle shape [11,14] and spindle/grain shape [12]; hence, they are indistinguishable from Cat B and fusolin crystals, respectively. Although another protein, i.e. human λLC, crystallizes in the ER, it produces various crystal morphologies at once [16]; thus, it is not suitable as a reporter for this assay. (iv) To circumvent this morphology limitation problem, an immunoglobulin M (IgM)-like multivalent secretory protein denoted as ‘scFv-Fc-stp (*N*>A)’ [13] had to be selected based on its characteristics to produce spherical protein droplets in the ER via LLPS (Figure 1D). This protein possesses cryoglobulin-like characteristics that render it to precipitate at temperatures lower than 37°C by forming protein droplets by LLPS [13]. Importantly, as shown by the immunofluorescent micrographs in Figure 1A–D, all four types of inclusion bodies were encapsulated in the calnexin-positive ER membranes, demonstrating the site of their phase separation is in the ER lumen.

### The co-expression of two cargo protein pairs in all six combinations demonstrates that there is no interference between any pairs of cargo proteins

The selected four model proteins were co-expressed in pairs in all six combinations. This two-gene co-expression study intends to validate that none of the four model cargo proteins prevent other proteins from reaching their respective threshold concentrations in the ER lumen. Likewise, the experiment ensures that no two protein cargo pairs are indistinguishable when two morphological types of inclusion bodies are produced side by side in the same continuous ER lumenal compartment.

**Figure 2:**
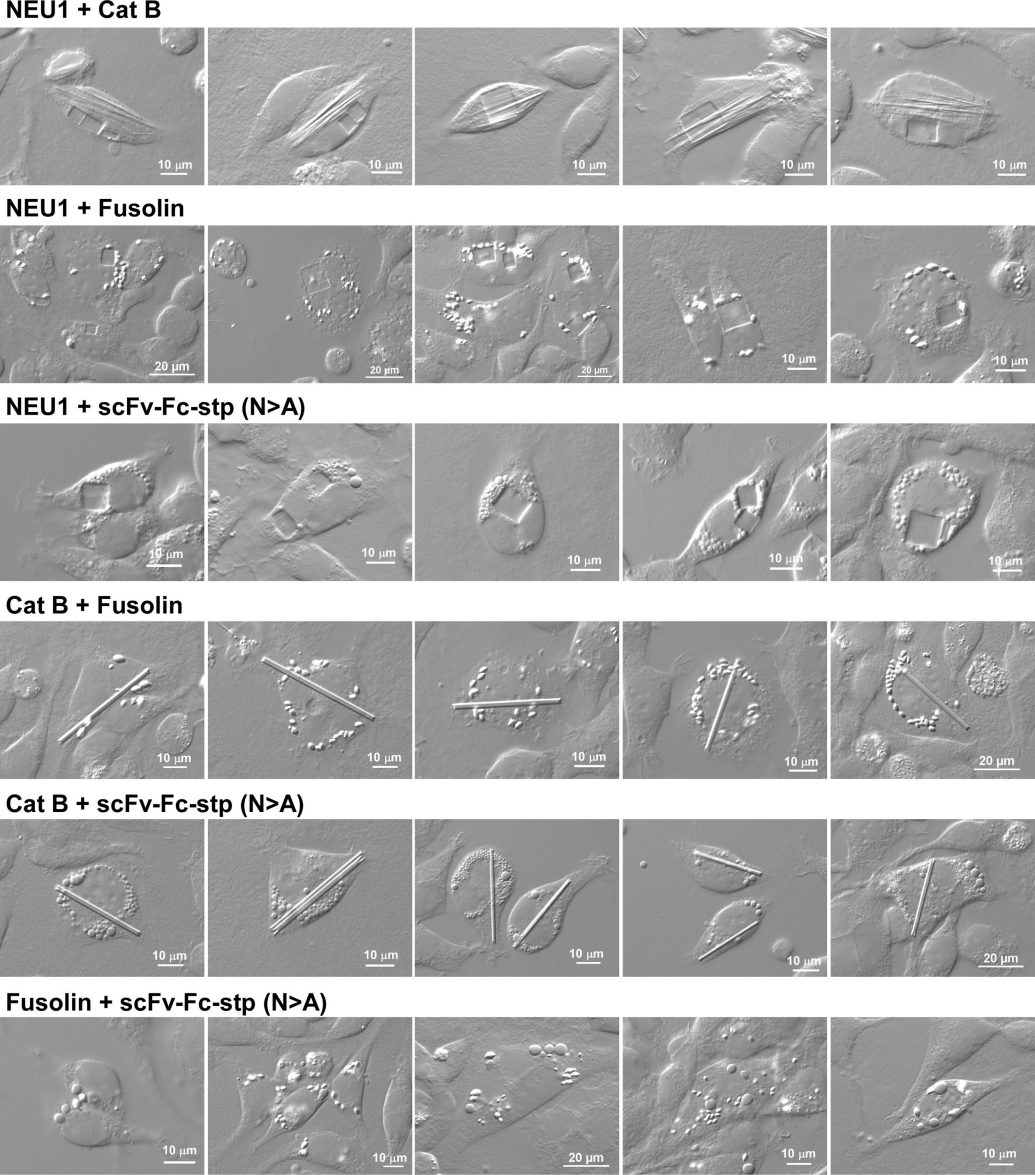
Cargo protein maintains the characteristic inclusion body morphology regardless of the co-expressed cargo proteins. Differential interference contrast (DIC) micrographs of transfected HEK293 cells co-expressing the two model cargo pairs in six combinations. On day-4 post-transfection, cells were fixed and imaged. Five representative image fields with the cells of interest are shown for each co-expression pair. Co-expressed protein names are shown on the left side of each row. Cat B, cathepsin B.

The two-cargo co-expression experiments demonstrated that individual inclusion body morphology native to each cargo protein was maintained even when co-expressed with another cargo protein in the same ER lumen (Figure 2). Although a thinning of the individual Cat B rod was observed when co-expressed with NEU1 (Figure 2, top row), there was no change in inclusion body morphology. Therefore, no two phase-separation events were mixed into generating ambiguous intermediate inclusion types. Even when two similar inclusion bodies—scFv-Fc-stp droplets and fusolin spindles—were formed side by side, they were readily distinguishable based on their appearances (Figure 2, bottom row). Overall, there were no instances where one protein phase-separation event prevented the phase separation of the other protein in the ER. In other words, respective model cargo proteins could find and associate with their own ‘mates’ in the crowded environment of the ER and produce characteristic inclusion bodies that coexist without mixing or interfering with one another.

These results indirectly suggested that no pairs of inclusion bodies triggered severe ER stress that shuts off cellular protein translation [43]. If any of the cargo pairs induced detrimental levels of ER stress, each cargo protein pair would not have synthesized to a high enough protein concentration to induce phase separation in the ER. Likewise, the expressing cells would not have maintained high viability to foster inclusion bodies during the course of the experiment [44].

### The co-expression of all four cargo proteins led to simultaneous phase-separation events in the ER

One of the main purposes of this study is to challenge cellular capacity and evaluate whether up to four different types of inclusion bodies can be induced and accommodated simultaneously in the ER lumen of a single cell. As noted above, the key to identifying each independent phase-separation process in concurrently happening four parallel events is assessing the inclusion body morphology that reports the protein identity.

**Figure 3:**
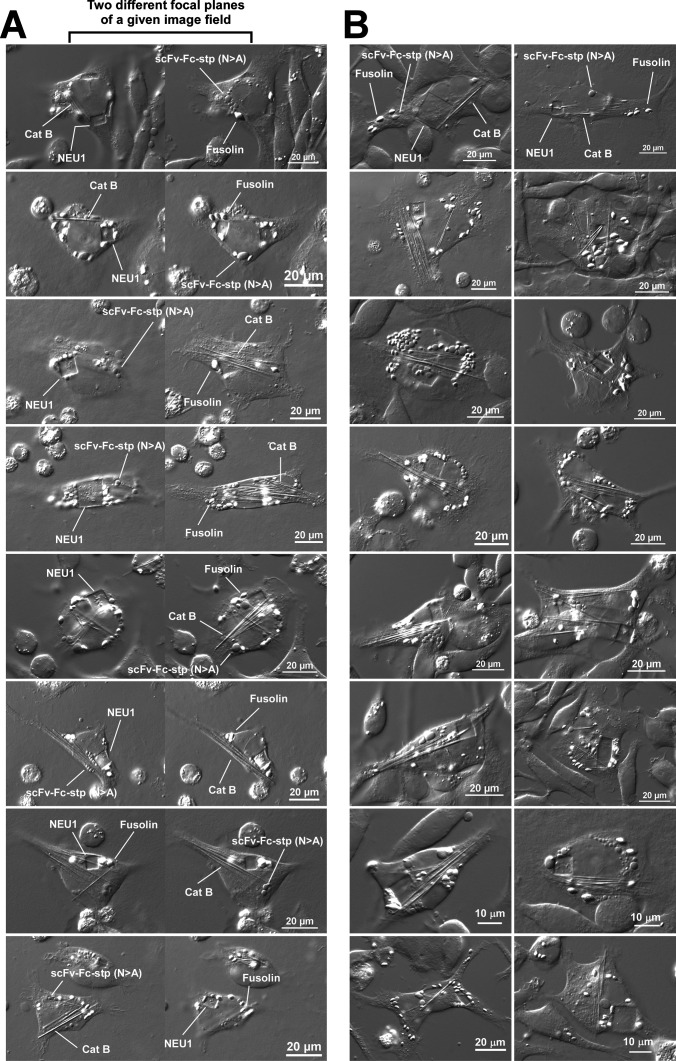
All four types of inclusion bodies coexist in the ER lumen without morphological mixing. Differential interference contrast (DIC) micrographs of HEK293 cells co-expressing four model cargo proteins simultaneously. On day-4 post-transfection, cells were fixed and imaged. (**A**) Representative eight-image fields captured at two adjacent focal planes are shown side by side. Readily distinguishable inclusion body examples are labeled. (**B**) Sixteen independent image fields showing the cells of interest that house all four inclusion body types in a single focal plane. Representative inclusion bodies are labeled in two images at the top row. Cat B, cathepsin B.

When all four expression constructs were co-transfected, ‘many’ transfected cells contained all four different inclusion body types at different efficiency and frequency by day-4 post-transfection, although it required a thorough visual scanning of various focal planes using high-power magnification to examine a given cell of interest one at a time. While the formation of all four distinct inclusion bodies could be confirmed inside the cells during microscopic observation, the micrographic documentation was not straightforward because different inclusion bodies were often present in different focal planes of a given cell and in different numbers and sizes, usually oriented in various directions within the cell. Therefore, quantitatively deriving the actual phenotype frequency of cells that housed all four different inclusion types was not feasible because some inclusion bodies are easily missed out even when each cell was carefully z-scanned from the tip of the cells all the way to the bottom of the cells in smallest increments. Nevertheless, numerous examples were documented for the cells that unquestionably housed all four inclusion body types within a depth of two neighboring focal planes. Although the numbers stated below do not have any statistical power because the determination of phenotype frequency was not possible, out of three repeats of transfection experiments, 30 independent pairs of two focal plane images (8 of them are shown in Figure 3A), and 72 independent single focal plane images (16 of them are shown in Figure 3B) were captured to record simultaneous phase separation of all four cargoes in single cells. The author’s intention to report these numbers is to illustrate that it is not a rare one-in-a-million event. For similar technical reasons, the size distribution of the four different inclusion bodies, as well as the precise number of each inclusion body type in a single cell, was difficult to determine because each of the four inclusion bodies was located in different focal planes of the cells in different numbers and densities, and their orientations varied greatly.

In Figure 3A, images taken from two adjacent focal planes are displayed side by side for eight representative fields showing the cells of interest that unquestionably house all four inclusion body types at once. Inclusion bodies that represent each of the four cargo proteins are pointed out in the image panels. The panels in Figure 3B are 16 independent image fields showing the cells of interest that house all four inclusion body types simultaneously in a single focal plane. Additional cellular images are provided in an accompanyingSuppl-1 to illustrate the diversity and complexity of inclusion body phenotypes. Even among the cells that housed all four inclusion bodies simultaneously, the number of inclusion bodies for each cargo varied significantly from cell to cell. Likewise, even within a single cell, the size difference of each cargo’s inclusion bodies varied tremendously. However, given that individual proteins spontaneously produced the telltale inclusion bodies without morphological mixing, inclusion bodies of each cargo protein were biochemically and spatially segregated from each other and coexisted in the ER lumen. Furthermore, transiently transfected HEK293 cells demonstrated sufficient biosynthetic capacity and membrane plasticity to house all four different types of inclusion bodies simultaneously in the ER lumen without compromising cell health.

## Discussion

Identifying four biologically and evolutionarily unrelated model cargo proteins made it possible to visualize the simultaneously occurring multiple phase-separation events in the ER lumen, as well as to challenge the limit of a cell’s morphological plasticity and cellular biosynthetic capacity. Co-transfected four cargo proteins independently accumulated in the ER lumen until reaching their respective threshold concentrations and produced characteristic mesoscale inclusion bodies by phase separation. It is fair to point out that the precise intracellular threshold concentration for each cargo would be difficult to show experimentally, but the fact that each of the four cargoes spontaneously phase-separated suggested that threshold concentration was reached even with one-quarter of plasmid gene dosage. Because the characteristic inclusion body morphology was preserved during the simultaneous phase-separation phenomena, the inclusion body’s identity was readily distinguishable even when all four phase-separation events occurred at once. None of the four cargo proteins inhibited the accumulation and phase separation of the other three proteins in the ER lumen, suggesting no dominant–recessive relationship among the four model cargo proteins used. While the precise state of ER stress levels for four-inclusion-containing cells was not possible to demonstrate, the fact that the crystals and droplets continued to grow in size and number over time clearly suggested that active protein translation sustained the growth of corresponding inclusion bodies. In other words, even if the ER stress might have been activated, the cells managed the stress without shutting off the protein translation machinery. This observation was clearly different from the accumulation of misfolded/unfolded proteins that induced Russell body-like inclusion bodies in the ER and suppressed cellular protein synthesis [15,44]. Furthermore, the fact that inclusion body morphology was not mashed up indicated that each cargo protein associates only with its mates to grow the size of respective inclusion bodies in the presence of numerous other proteins in a highly crowded ER lumen. Lastly, the ER membrane is flexible enough and perhaps expandable on demand to accommodate four distinct inclusion body types simultaneously without damaging organellar integrity and compromising cell health. The question is whether four different inclusion bodies are the limit of biosynthetic capacity and membrane plasticity. From the viewpoint of protein science and material science, it is an important topic to explore how many different types of inclusion bodies are producible simultaneously in a single cell or an organelle. However, such an experiment is currently limited by the availability of cargo proteins that produce distinguishably unique inclusion body morphology.

In the cases of membrane-less organelle formation by LLPS, functionally antagonizing condensates made of different protein constituents can sometimes coalesce and mix in the cytoplasm to regulate the functions of respective condensates, as shown for the Hippo signaling pathway [45]. By contrast, demixing was the process for membrane-less organelle formation at postsynaptic densities in neurons, where excitatory and inhibitory condensates spontaneously phase-separated into distinct sub-compartments in dendritic spines [46]. Yet in another example, the nucleolus is shown to be a multi-phase condensate comprising three internal liquid phase-separated sub-compartments where different proteins are enriched in each sub-compartment [47]. Now, unlike those proteins that physiologically function in the same pathway or compartment, the four model cargoes used in this study are biologically, evolutionarily, and functionally unrelated to each other; thus, there were no known protein–protein interactions or sequence homology among them. They are also from different species or even derived from a synthetic sequence. Because they do not naturally function together or coexist in the ER lumen of the human cells, the current findings do not directly provide insights into the process of physiological or pathological phase-separation events. However, the fact that cargoes were selected solely based on their inclusion body morphology (regardless of their origins) turned out to be beneficial in visualizing the simultaneously occurring four different phase-separation events in the ER lumen. In theory, when the protein self-association force is stronger than the random protein–protein interactions, repulsion, or diffusion in the ER, each model cargo (having a strong tendency to self-associate) would spontaneously phase-separate into inclusion bodies after reaching the threshold concentrations, instead of maintaining homogeneously mixed conditions. Evidently, there seem to be no significant protein−protein interactions other than the self-association for each model cargo. In fact, without taking advantage of these four cargoes, the visualization of four concurrent events may not have been even possible. It is currently unknown whether similar modes of inclusion body demixing widely apply to other sets of phase-separating proteins in the ER lumen if the participating proteins have co-evolved to play certain physiological roles together or have some affinities to one another. Furthermore, it still remains unknown whether it is possible (and if it is, then under what conditions) that two or more distinct crystal inclusion bodies can morphologically mix or merge.

## Future directions

Similar studies can be designed by selecting a set of crystallizing cargoes that (i) have sequence homology, (ii) are known to interact with each other, or (iii) are co-evolved to function in the same pathway. Unlike the current study that purposefully employed evolutionarily unrelated model cargoes, such study design should uncover new insights into the intracellular protein mixing and demixing process that mimics physiological and pathological settings.

## Materials and methods

### Detection antibodies and reagents

Rabbit anti-calnexin (cat. C4731) was from Sigma-Aldrich. Rabbit anti-giantin was from Covance (cat. PRB-114P). Mouse anti-LAMP1 (clone H4A3, cat. sc-20011) was from Santa Cruz Biotechnology. All chemical reagents were obtained from Sigma-Aldrich unless specifically mentioned.

### Expression constructs

The expression constructs encoding human NEU1 [15], ACEV fusolin [16], and scFv-Fc-stp (*N*>A) [13] were previously reported. The nucleotide sequence for *T. brucei* Cat B was generated from the amino acid sequence UniProt D6XHE1 after optimizing the codon usage to human preference by using a publicly available tool from GENEWIZ (South Plainfield, NJ, U.S.A.). The recombinant DNA sequence was verified by the Sanger method and seamlessly cloned into a pTT^®^5 mammalian expression vector licensed from the National Research Council of Canada.

### Cell culture and transfection

HEK293-6E cell line (herein HEK293) was obtained from the National Research Council of Canada. HEK293 cells were suspension cultured in a humidified CO2 Reach-In incubator (37°C, 5% CO2) using FreeStyle™ 293 Expression Medium (Thermo Fisher Scientific). The cells were grown in vented cap Corning^®^ Erlenmeyer flasks placed on Innova 2100 shaker platforms (New Brunswick Scientific) rotating at 130‒135 rpm. The expression construct was transfected into HEK293 cells using a commonly used polyethyleneimine method. Difco yeastolate cell culture supplement (BD Biosciences) was added to the suspension cell culture at 24-h post-transfection. In a two-gene transfection experiment, a 1:1 plasmid ratio was used without changing the total amount of DNA transfected. Likewise, in four-gene transfection, 1:1:1:1 DNA ratio was used while keeping the total amount of DNA.

### Microscopy and cell phenotype screening

Transfected cells in suspension format were seeded onto poly-d-lysine-coated glass coverslips at 48-h post-transfection. Cells were then statically cultured up to day-6 post-transfection in CO2 incubators at 37°C. At a designated time after transfection, cells were fixed in 100 mM sodium phosphate buffer (pH 7.2) containing 4% paraformaldehyde for 30 min at room temperature. Fixed cells were directly used for differential interference contrast (DIC) microscopy or processed for indirect immunofluorescent microscopy. For immunostaining, cells were permeabilized in phosphate-buffered saline containing 0.4% saponin, 1% bovine serum albumin, and 5% fish gelatin for 15 min, followed by incubation with primary antibodies for 60 min. After three washes in the permeabilization buffer, the cells were probed with secondary antibodies for 60 min in the permeabilization buffer. Coverslips were mounted onto slide glass using Vectashield mounting media (Vector Laboratories). The slides were analyzed on a Nikon Eclipse 80i microscope with a 60× or 100× CFI Plan Apochromat oil objective lens and Chroma FITC-HYQ or Texas Red-HYQ filter. DIC and fluorescent images were acquired using a Cool SNAP HQ2 CCD camera (Photometrics) and Nikon Elements BR imaging software. To identify cells that house all four types of inclusion bodies, the fields of cells were manually scanned systematically using a high-power objective lens by carefully changing the plane of focus and capturing cell images one field and one focal plane at a time. As shown in Figure 3 and Supplement-1, due to the complexity of four-inclusion-containing cell morphology, the four-inclusion-positive cell frequency, inclusion size distribution for each protein, and the number of each inclusion body per cell were not able to determine in this study.

## Supplementary material

Online supplementary figure 1

## Data Availability

The author confirms that the data supporting the findings of this study are available within the article and its supplementary material.

## Abbreviations

ACEV: *Anomala cuprea* entomopoxvirus
Cat B: cathepsin B
DIC: differential interference contrast
ER: endoplasmic reticulum
IgM: immunoglobulin M
LAMP1: lysosome-associated membrane glycoprotein 1
LLPS: liquid–liquid phase separation
NEU1: neuraminidase 1
