# Supplementary material for: Demixing of four simultaneously co-expressed phase-separating proteins in the endoplasmic reticulum lumen
Source: Biosci Rep. 2025 Sep 2;45(9):481–90. doi: 10.1042/BSR20253165 (PMC12599291; doi:10.1042/BSR20253165)

**Demixing of four simultaneously co-expressed phase-separating proteins in the endoplasmic reticulum**

Haruki Hasegawa *

**Supplement 1. All four distinct inclusion bodies coexist in the ER lumen.**

(A, B) Representative four image fields captured at two adjacent focal planes (A) or three neighboring focal planes (B) are shown side-by-side. (C) Thirty-five independent image fields showing the cells of interest that house all four detectable inclusion body types in a single focal plane.


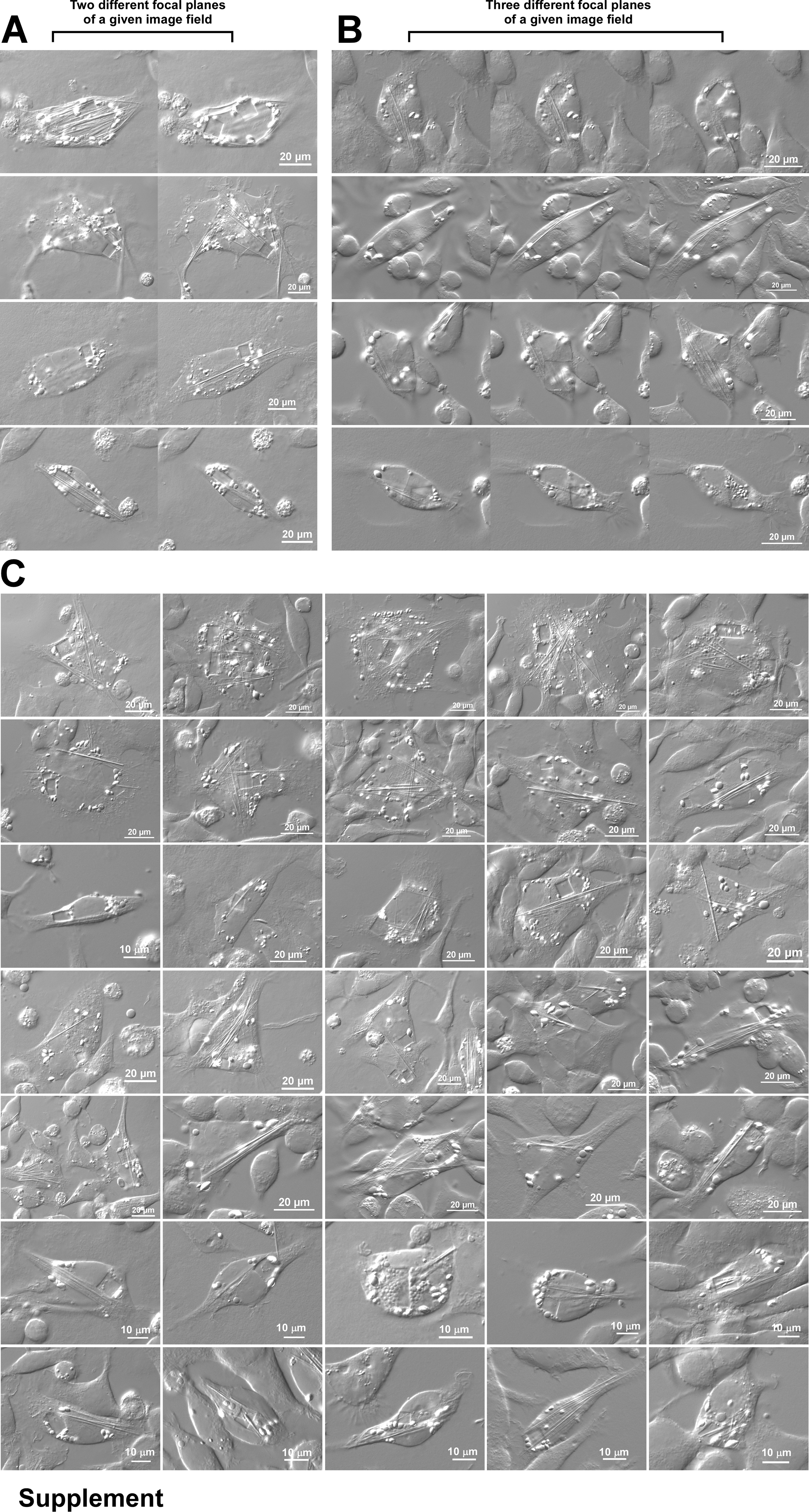

Supplement: Online supplementary figure 1 [file bsr-45-09-BSR20253165-s001.docx]
